# Supplementary material for: Predictors of COVID-19 vaccine uptake among persons aged 18 years and above in Ga North Municipality, Ghana using the Health Belief Model: A community-based cross-sectional study
Source: PLoS One. 2023 Nov 7;18(11):e0293350. doi: 10.1371/journal.pone.0293350 (PMC10629641; doi:10.1371/journal.pone.0293350)
Supplement: Attachment — Submitted filename: PloS One response to review comments.docx [file pone.0293350.s002.docx]

**RESPONSE TO REVIEWERS**

***Reviewer 1***

The manuscript is well written but there are some minor corrections.

**Comment**

I suggest that the authors rewrite the background to reflect the variables that were studied.

**Response**

Thank you for this comment. The background has been rewritten to reflect the variables that were studied.

**Comment**

The authors wrote about vaccine hesitancy at the third paragraph and had little write-up as it relates to factors influencing vaccine uptake.

**Response**

Thank you for this comment. This has been addressed.

**Comment**

In addition, the writers wrote extensively on health care workers when they are not the main population.

**Response**

This comment has been addressed.

**Comment**

There was a spelling mistake in the last sentence before the conclusion, the letter 'e' is missing from the predictors. I suggest that you change the statement the population of the non-vaccinated people is high because the study did not cover the number of people who are vaccinated, the study only covered uptake. Furthermore, the percentage of the uptake is high. Therefore, I suggest that it is changed.

**Response**

Thank you very much for these important comments. The issues raised have been duly addressed.

***Reviewer 2***

**Comment**

All relevant data should be included in the study. Avoid duplication of data.

**Response**

Thank you for this comment. This has been addressed; all relevant data have been included as an appendix

**Comment**

The non-vaccinated group should be statistically identified. Studies can also be conducted on the other groups.

**Response**

Thank you for this comment. The non-vaccinated group made up 27.8% of the study population statistically. We agree with your comment that further studies can also be conducted on them and other groups subsequently.

**Comment**

Health promotion interventions should can be extended further to eradicate barriers in taking covid-19 vaccines and other related vaccines.

**Response**

Thank you very much for this comment. Our study determined the predictors of COVID-19 vaccine uptake based on the constructs of the Health Belief Model, of which barriers are part. From our findings, barriers were among the significant predictors of vaccine uptake and recommendations were made in this regard. Studies can be conducted regarding the uptake of other vaccines if there is a need for that.

***Reviewer 3***

**General comments**

I have had the privilege of reviewing your manuscript. The study was well-conceptualized, conducted and written. The use of the health belief model was also appropriate. I have a few critiques for you to consider to improve the quality of the write-up to meet international readability standards.

Thank you for the good study!

**Response**

The authors are appreciative of your kind words and commendation. Thank you for the review. The authors have duly acknowledged and addressed your comments.

**Comments**

The manuscript will benefit from a general review to mitigate grammatical and syntax errors.

**Response**

Grammatical errors have been corrected.

**Comment**

The study background seems rather lengthy. Could it be more precise? Focusing the write-up entirely on the subject matter (COVID-19 vaccine uptake) may help.

Response

Thank you very much for this comment. We have addressed the issue.

**Comments**

Since this is a cross-sectional study, why did you require the participants to intend to live in the municipality to be eligible? The description of the study population seems more like the inclusion criteria, which is then repeated immediately after.

**Response**

The comments have been noted and duly addressed.

**Comment**

The formula you use for sample size determination has been attributed to another set of authors. This is the first time I am seeing it attributed to Degu and Tessema formula. Kindly verify this and adjust if necessary.

**Response**

Thank you so much for this very important comment, which has now been addressed.

**Comments**

What motivated the choice of 5% as the non-response rate?

**Response**

This was chosen because of the sensitive nature of the study, which could lead to an increased dropout rate of the participants. 5% has been used in a previous study that I have now cited.

**Comment**

Why were the sample sizes disproportionately allocated among selected communities? Did you mean to say proportionately allocated?

**Response**

This has been addressed.

**Comment**

What is your outlook on the coefficient for internal consistency (alpha Cronbach?) less than 0.7? How does this affect the interpretation of the perceived severity and benefit domains? Could these values have been improved by dropping some of the items in the domains?

**Response**

The overall alpha coefficient for the entire HBM was 0.8554 which is excellent. The coefficients were calculated using the best combination of items under each construct of the HBM, excluding the response options. Cronbach's alpha does not provide a very good estimate when the items making up the measurement scale are small in number. The more items to measure a construct the more accurate the coefficient. Therefore, the relatively low alpha estimated for perceived severity (0.6759), may be due to the small number of items measuring this construct (3 items).

**Comment**

You had a 100% response rate with no missing data. Are there specific lessons other researchers (especially in the sub-Saharan African context) could learn to help achieve these? It is critical to underscore this detail in the study.

**Response**

This response rate was achieved possibly because the purpose of the study was duly explained to the respondents and the principal investigator and the research assistant were present to clarify issues and provide answers to questions posed by the respondents during data collection.

**Comment**

You used relevant literature quite well but could also consider other regional findings from studies like https://doi.org/10.1371/journal.pone.0267691.

**Response**

This point has been noted and the recommended reference and others have been included in a subsequent paper that we are drafting. The current study focused on vaccine uptake and its predictors based on the Health Belief Model. The recommended paper above was a rapid review of papers published on COVID-19 vaccine acceptance and associated factors in Nigeria. There is a difference between vaccine acceptance and vaccine uptake. The paper would fit well in the manuscript we are drafting.
